# CSRP1 Promotes Colon Adenocarcinoma Growth and Serves as an Independent Risk Biomarker for Worse Prognosis

**DOI:** 10.1155/2023/8586507

**Published:** 2023-04-18

**Authors:** Senlong Yu, Haifeng Zhao, Hongjie Meng, Shengguang Shi, Shenghui Cao, Tianhua Bian, Canping Ruan

**Affiliations:** ^1^Department of Gastrointestinal Surgery, Zhuji People's Hospital of Zhejiang Province (Zhuji Affiliated Hospital of Shaoxing University), Zhuji 311800, China; ^2^Department of General Surgery, Zhuji Chinese Traditional Medical Hospital, Zhuji 311800, China; ^3^Colorectal Surgery Department, Changzheng Hospital, Shanghai 200003, China

## Abstract

**Background:**

Cysteine and Glycine Rich Protein 1 (CSRP1) belongs to the cysteine-rich protein family, which contains a unique double-zinc finger motif and is important for development and cellular differentiation. Abnormal expression of CSRP1 was reported within several malignancies such as prostate cancer and acute myeloid leukemia. Here, we explored function of CSRP1 within colon adenocarcinoma (COAD) for the first time.

**Methods:**

The mRNA levels of CSRP1 in COADs were obtained from TCGA datasets. CSRP1 protein expressions in COADs were tested via immunohistochemistry staining. Patients' prognosis was evaluated using both univariate analysis and multivariate analysis. Two human COAD originated cancer cell lines, Caco-2, and HT-29, were used for cellular experiments including shRNA knockdown, proliferation assay, and migration assay. In vivo model was established using nude mice xenografts to further validate the role of CSRP1 in COAD progression.

**Results:**

The mRNA levels of CSRP1 are elevated in COAD specimens from patients with more advanced tumor stages and higher Carcinoembryonic Antigen (CEA) levels. In addition, higher CSRP1 mRNA level indicates worse COAD prognosis. Consistently, higher CSRP1 protein expression is correlated with worse overall survival according to both univariate and multivariate analysis, indicating that CSRP1 is a new COAD prognostic factor. Furthermore, COAD cells transfected with CSRP1-shRNAs exhibit attenuated proliferation and migration capacities. Finally, growth of xenografts originated from CSRP1-knockdown cells is inhibited comparing to the control ones.

**Conclusions:**

Expression of CSRP1 is positively correlated with COAD progression, which can promote tumor growth and migration. Higher CSRP1 can is a novel independent prognostic factor of COAD.

## 1. Introduction

The cDNA encoding Cysteine and Glycine Rich Protein 1 (CSRP1) was initially reported in 1990s, which is a highly conserved serum-inducible immediate early response gene. Human CSRP1 gene is located in chromosome 1q24–1q32 [1]. The CSRP1 expression can be quickly induced upon serum repletion in serum-starved cells, highlighting its potential role in cell growth and cell cycle [2]. For example, CSRP1 is highly expressed in smooth muscle cells during embryogenesis [3]. CSRP1-knockout mice exhibited decreased neointima formation after arterial injury [4]. Besides, it participates in spinal cord repairmen after injury in zebrafish [5]. Mechanically, CSRP1 can bind with actin, Dishevelled and Diversin, therefore participates in cytoskeletal organization. Disruption of CSRP1 signaling leads to abnormal convergent extension of cell movement [6]. In addition, CSRP1 has been reported to participate in cell differentiation. For example, stimulation of human fetal femur-derived cells with fibroblast growth factor (FGF)-2 resulted into a significantly decreased CSRP1 level and reduced amino acid turnover, suggesting an undifferentiated cell status [7].

Furthermore, abnormal expression of CSRP1 was identified in several tumors. On one hand, decreased CSRP1 protein expression was observed in hepatocellular carcinoma (HCC) [8], indicating its role as an anticancer factor. Similarly, CSPR1 is decreased in prostate cancer tissues compared to that in normal prostate tissues, and lower CSRP1 can predict a better disease-free survival of prostate cancer [9].

In contrast, higher CSRP1 expression was reported in breast cancers, which is related to RNA-binding functions [10], suggesting its oncogenic potentials. Consistently, higher expression of CSRP1 helps identify a high-risk subgroup of acute myeloid leukemia (AML) with worse prognosis [11]. Therefore, CSRP1 seems to display completely different roles in different tumor types. A previous study also suggested the dysregulated expression of CSRP1 in colon adenocarcinoma (COAD) [12], however, the study did not further investigate its clinical significance or functional mechanisms. Therefore, here we systematically mapped the expression, clinical relevance, and functions of CSRP1 in COAD from clinical, cellular, and in vivo aspects.

## 2. Methods

### 2.1. Online Datasets and Analyses

The mRNA level of CSRP1 in COAD was extracted from TCGA datasets and compared in the form of fragments per kilobase of exon per million (FPKM). The basic characteristics including *T* stage, *N* stage, *M* stage, lymphatic invasion, serum Carcinoembryonic Antigen (CEA) level, and survival information were all retrieved from the datasets.

### 2.2. Patients' and Samples

Totally 167 COAD tissues were retrospectively collected from Zhuji People's Hospital of Zhejiang Province. All tissue specimens were formalin-fixed and paraffin-embedded (FFPE). Patients with previously other malignancies (except thyroid cancer) were excluded.

### 2.3. Immunohistochemistry (IHC) Staining

IHC staining was conducted to assess protein expression levels of CSRP1 in COAD tissue specimens as we previously reported [13]. FFPE samples were cut into 4 *μ*m sections and treated with standard IHC procedures with the following sequence: dried, deparaffinized, antigen retrieval, blockage, primary antibody incubation, secondary antibody incubation, and 3,3 Diaminobenzidine (DAB) staining.

### 2.4. Cell Culture and Knockdown

Caco-2 and HT-29 cell lines were purchased from American Tissue Culture Collection (ATCC). All cells were maintained in Ham's F-12 medium supplemented with 10% FBS [14]. The shRNAs were purchased from Thermo Fisher Scientific (Pittsburgh, PA, USA), including two specific shRNAs targeting human CSRP1 (shRNA#1 and shRNA#2) as well as a nonspecific scrambled control shRNA. CSRP1 knockdown was achieved by shRNA infection following the manufacture's procedure.

### 2.5. Western Blot (WB)

Protein expression of CSRP1 in cultured cells was tested via WB. Extracted proteins from cells were firstly subjected to SDS-PAGE electrophoresis, followed by transferring to polyvinylidene difluoride (PVDF) membranes, primary antibody incubation, secondary antibody incubation, and chemiluminescent (ECL) detection [15].

### 2.6. Proliferation Assay

Proliferation capacities of Caco-2 and HT-29 cells were tested by Cell Counting Kit-8 (CCK-8) method following manufacture's procedures in 96-well plates [16]. Briefly, the cell seeding number was 5000 cells/well and cultured in Ham's F-12 medium supplemented with 10% FBS. The absorbance at OD 450 nm was tested after culturing for 1, 2, 3, and 4 days, respectively. All the experiments were conducted in triplicates.

### 2.7. Migration Assay

Transwell method was used to evaluate cell migration capacity [17]. About 5000 transfected cells were seeded into the upper chamber of the Transwell insert (Corning, Cambridge, MA, USA). Cells were cultured in 5% CO_2_ at 37°C for 48 h, and then the Transwell inserts were taken out to fix and stain the cells on the upper surface of chambers. Migrated cells were counted.

### 2.8. Mice Model

BALB/c nude mice were obtained from the Shanghai Laboratory Animal Center (SLAC, Shanghai, China). Briefly, mice xenograft model was generated by subcutaneously injecting transfected COAD cells into the nude mice. After one month, subcutaneous mice xenografts were resected to weight and picture.

### 2.9. Statistics

The SPSS software was used for data analysis. Statistical significance was confirmed by Student's *t*-test for cellular and animal experiments, Chi-square test for clinical data analyses, and Kaplan–Meier test and Cox hazard regression test for survival analyses. Overall survival (OS) is defined as the time from treatment to death, regardless of disease recurrence. Disease-free survival (DFS) is defined as the time from treatment to recurrence of tumor or death. Progression-free survival (PFS) refers to the length of time during and after the treatment cancer, that a patient lives with the disease but it does not get worse.

### 2.10. Ethics

Written informed consent was obtained from each participant. The Research Ethics Committee of Zhuji People's Hospital of Zhejiang Province reviewed and approved all protocols of this study.

## 3. Results

### 3.1. CSRP1 mRNA Level Is Positively Correlated with Advanced COAD Characteristics

**Figure 1:**
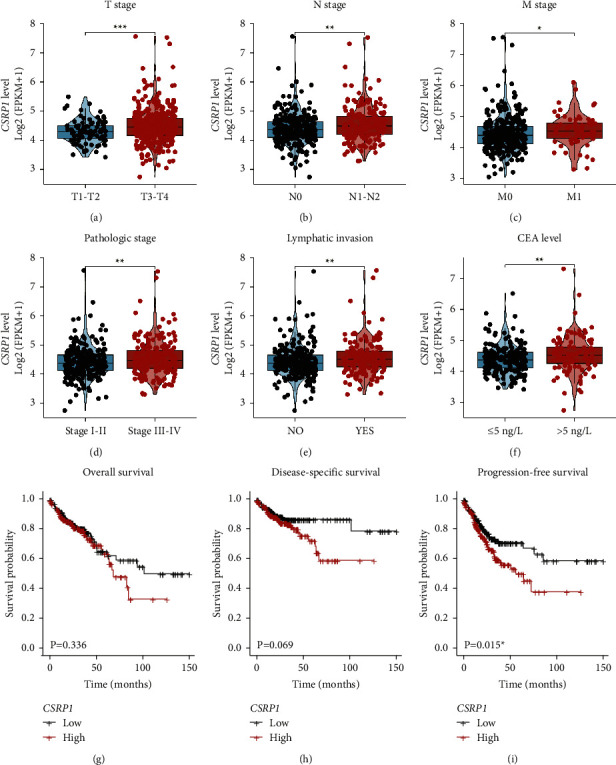
The mRNA level of CSRP1 in TCGA-COAD datasets and its clinical significance. The mRNA level of CSRP1 in COAD tissues was extracted from TCGA dataset. By unpaired student's *t*-test, we found that CSRP1 was positively correlated with COAD *T* stage (a), *N* stage (b), *M* stage (c), TNM stage (d), lymphatic invasion (e), and CEA level (f) In addition, we subgrouped TCGA patients into low-CSRP1 group and high-CSRP1 group to further assess its effect on COAD overall survival (g), disease-free survival (h), and progression-free survival (i), respectively.

We firstly retrieved the information of CSRP1-mRNA in COAD tissues from TCGA datasets. According to statistically analyses, we found that patients with higher *T* stage showed elevated CSRP1-mRNA levels in their tumor specimens (Figure 1(a), *P* < 0.001). Similarly, CSRP1-mRNA levels were higher in COAD cases with positive lymph nodes (Figure 1(a), *P* < 0.01) and distant metastases (Figure 1(c), *P* < 0.05). Consistently, patients with TNM stage III-IV showed higher CSRP1-mRNA levels than those with TNM stage I-II (Figure 1(d), *P* < 0.01). Besides, lesions with positive lymphatic invasion showed higher CSRP1-mRNA levels than those without lymphatic invasion (Figure 1(e), *P* < 0.01). Similar observation was found on that CSRP1-mRNA level was positively correlated with serum CEA level (Figure 1(f), *P* < 0.01).

### 3.2. High CSRP1 mRNA Level Indicates Unfavorable COAD Prognosis from TCGA Datasets

Considering that all the above-given factors were well-recognized prognostic factors of COAD, we speculated a possible prognostic role of CSRP1-mRNA in COAD. Although the TCGA datasets did not identify any significant effect of CSRP1-mRNA on the overall survival (Figure 1(g), *P*=0.336) or disease-free survival (Figure 1(h), *P*=0.069) of COAD, patients with higher CSRP1-mRNA levels tend to exhibited worse prognoses especially since 5-years postdiagnosis. Moreover, CSRP1-mRNA level was negatively correlated with COAD progression-free survival as shown in Figure 1(i) (*P*=0.015), implying the involvement of CSRP1 in COAD.

### 3.3. Patients' Information and CSRP1 Protein Expression Pattern in Our Retrospective Cohort

Based on the in silico findings from TCGA datasets, we were engaged to further explore clinical meaning of CSRP1 protein in colon cancer. Therefore, we retrospectively enrolled 167 COAD patients from our hospital (Table 1). There were 86 females and 81 males. The median age at diagnosis was 66.0 years old, ranging 26–86 years old. Among them, 43 cases showed tumor location in ascending colon, 30 cases in transverse colon, 19 cases in descending colon, and the other 75 cases in sigmoid colon. The median tumor size was 3.5 cm in diameter. There were 33 cases with pathological *T* stage *T*1, 21 cases with *T*2, 91 cases with *T*3, and the other 22 cases with *T*4. Meanwhile, there were 71 cases identified with positively lymph nodes metastases, while the other 96 cases with negative lymph nodes. Among them, 68 cases received postoperative chemotherapeutic treatment, while the other 109 cases did not accepted or unknown.

**Figure 2:**
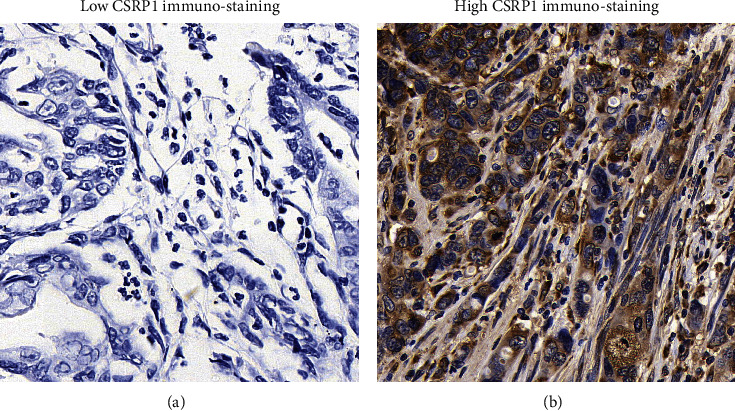
Protein expression of CSRP1 in COAD tissues. Representative IHC staining results of low-CSRP1 protein expression (a) and high-CSRP1 protein expression (b) images. Magnification: 400×.

By conducting IHC analyses of the tissue samples above, we found that CSRP1 protein exhibited different expression levels in different specimens (Figures 2(a) and 2(b)). Therefore, we further divided the 167 cases into low-CSRP1 protein expression group (*n* = 90) and high-CSRP1 protein expression group (*n* = 77) according to their immunostaining results. Chi-square tests revealed that patients in high-CSRP1 protein expression group exhibited larger tumor size (*P*=0.003) and higher possibility of lymph nodes metastases (*P*=0.049).

### 3.4. High CSRP1 Protein Expression is a Novel Independent Prognostic Factor

**Figure 3:**
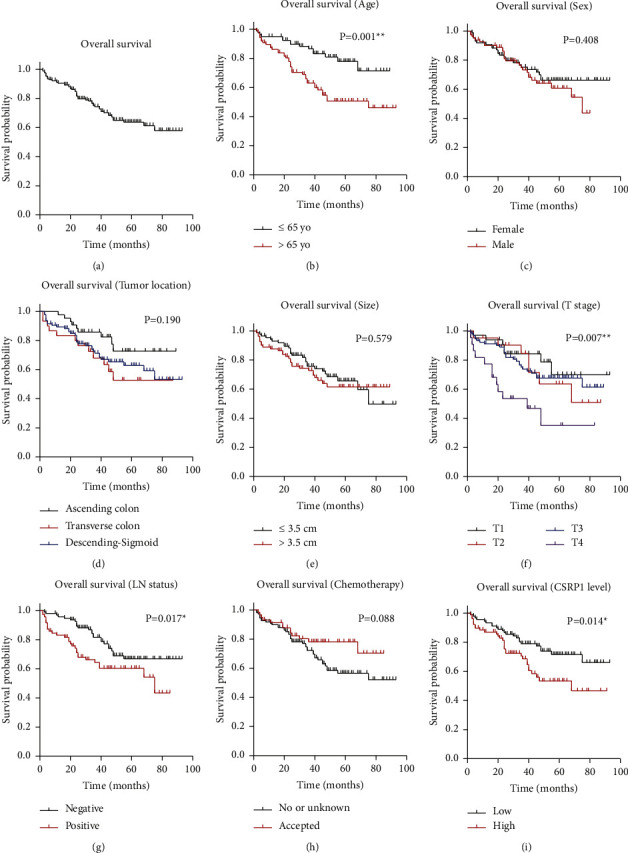
Survival analyses of COAD cohort from our hospital. The overall survival curve of entire COAD cohort was plotted using Kaplan–Meier method (a) In addition, survival curves were compared in different sub-groups divided based on patients' age (b), sex (c), tumor location (d), tumor size (e), *T* stage (f), lymph node metastasis (g), chemotherapy (h), and CSRP1 protein level (i)

We next performed survival analyses for all the 157 retrospectively enrolled COAD cases (Table 2). Till the last date of followup, 53 cases dead, and the 5-year overall survival (OS) rate was 63.7% (Figure 3(a)). Patients with elder age exhibited worse OS than younger ones (Figure 3(b), *P*=0.01). The 5-year OS rates of female patients and male patients were 66.2% and 60.7%, respectively, (Figure 3(c), *P*=0.408). The 5-year OS rates of patients with tumor location in ascending colon, transverse colon, descending-sigmoid colon were 72.7%, 52.7%, and 62.9%, respectively (Figure 3(d)). Although patients with ascending colon tumor location seemed to have better prognosis, the difference didn't reach statistically significance (*P*=0.190). Our data did not find any significant prognostic effect of tumor size (Figure 3(e), *P*=0.579), while the 5-year OS rate was 4% lower in patients with larger tumor size (61.4% vs. 65.7%). As expected, patients with advanced *T* stages exhibited worse prognosis than those with earlier *T* stages (Figure 3(f), *P*=0.007). For example, patients with stage *T*1 showed a 5-year OS rate of 70%, while *T*4 showed the 5-year OS rate as 35.2%. Meanwhile, patients with positive lymph nodes metastases had worse OS than those with negative ones (Figure 3(g), *P*=0.017). In our cohort, patients accepted chemotherapy showed better prognosis than those absent of chemotherapy (78.1% vs. 56.6%), although the difference was not statistically significant in univariate analysis (Figure 3(h), *P*=0.088). Of note, patients with higher CSRP1 protein levels had a significantly lower OS rate (53.3%) than those with lower CSRP1 protein levels (71.6%, *P*=0.014). The average survival time was 73.4 ± 3.5 months in low-CSRP1 group, while was only 58.7 ± 4.4 months in high-CSRP1 group (Figure 3(i)).

To exclude bias and confounders, we further conducted multivariate analysis. The variables in the model included patients' age, *T* stage, lymph node status, chemotherapy, and CSRP1 protein expression level (Table 3). Accordingly, elder age (HR = 2.489, 95% CI 1.337–4.632, *P*=0.004), *T*4 stage (HR = 3.108, 95% CI 1.123–8.600, *P*=0.001), and positive lymph nodes (HR = 2.853, 95% CI 1.501–5.424, *P*=0.001) all contributed independently to worse COAD prognosis. In contrast, patients accepted chemotherapy was an independent favorable prognostic factor (HR = 0.339, 95% CI 0.165–0.695, *P*=0.003). In addition, our data for the first time identified that higher CSRP1 protein expression was an independent unfavorable prognostic biomarker for COAD (HR = 1.895, 95% CI 1.078–3.330, *P*=0.026).

### 3.5. CSRP1-Knockdown Inhibits Proliferation and Migration of COAD Cells

**Figure 4:**
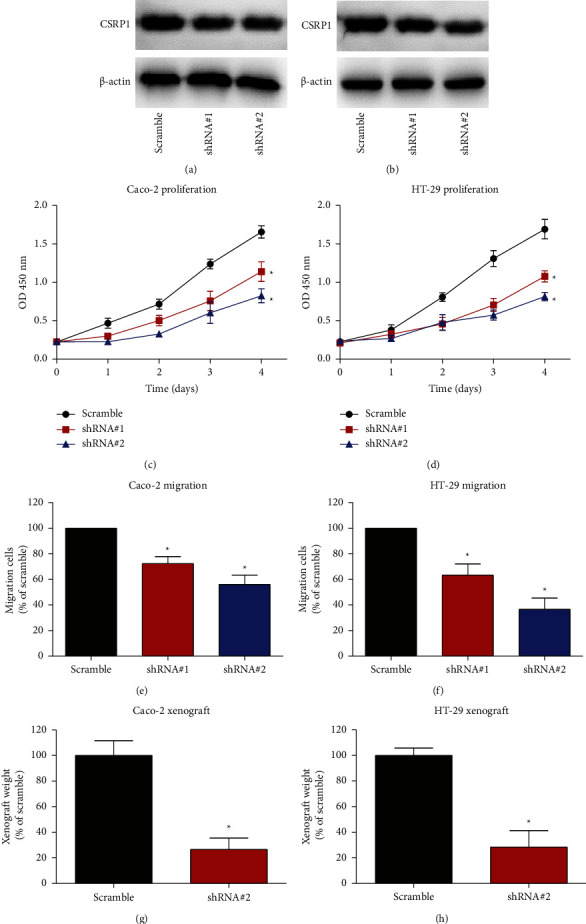
CSRP1-knockdown attenuates COAD progression both in vitro and in vivo. (a), (b) The shRNAs targeting CSRP1 and scrambled control shRNA were transfected into caco-2 and HT-29 cells, respectively. The knockdown efficiency was tested via western blot. (c), (d) CCK-8 assay was conducted to evaluate the proliferation curve of transfected cells. (e), (f) Transwell assay was conducted to assess the migration capacity of transfected cells. (g), (h) The xenografts originated from different transfected cell lines were weighted and compared after culturing for one month.

Since clinical data analyses revealed the important role of CSRP1 in COAD, we next aimed to validate its detailed effects through in vitro and in vivo experiments. The knockdown efficiency of shRNA#1 was not good in either Caco-2 (Figure 4(a)) or HT-29 (Figure 4(b)) cell lines compared to those transfected with scrambled control shRNA. However, the shRNA^#^2 results in 63% and 72% decreases of CSRP1 protein expression in Caco-2 (Figure 4(a)) or HT-29 (Figure 4(b)) cell lines, respectively. Phenotype analyses indicated that CSRP1-knockdown significantly attenuated COAD cell proliferation (Figures 4(c) and 4(d)) and migration (Figures 4(e) and 4(f)) capacities according to CCK-8 and Transwell results, respectively.

### 3.6. CSRP1-Knockdown Results in Attenuated COAD Growth in Mice Models

Finally, we established a xenograft COAD model using nude mice to provide more data on the tumor-correlated effects of CSRP1. One month after subcutaneous injection of transfected cells, the xenografts were resected (Figure 4(g)). Consistent with cellular data, the xenografts originated form CSRP1-knockdown cells showed significantly smaller tumor size and lighter tumor weight. Therefore, we came to the final conclusion that silencing CSRP1 inhibited COAD growth.

## 4. Discussions

According to a previous microarray data [12], CSRP1-mRNA level was decreased in 78.9% (15/19) COAD tissues comparing to adjacent colon tissues. Therefore, Zhou and his colleagues suggested that CSRP1 might suppress COAD [12]. Nevertheless, their study did not further compare the relationship between CSRP1 level and COAD characteristics. The study also lacks validation regarding the detailed tumor-related effects of CSRP1 in COAD cells. Here, in the current study, we not only analyzed the clinical relevance between CSRP1 and COAD survival, but also validated its tumor-promoting effect through cellular and mice experiments.

Our study has several limitations. Firstly, the mRNA level of CSRP1 was analyzed within TCGA datasets, while its protein level was analyzed in another cohort from our hospital. Although both its mRNA and protein levels showed the same prevalence on predicting unfavorable survival, further validation should be conducted in the same cohort. Secondly, the underlying functional mechanisms of CSRP1 in COAD progression remain unclear, further biochemical and biological studies will be necessary to provide more details. Thirdly, our study did not investigate the upstream mechanism of dysregulated CSRP1 in COAD. One inspiration is the methylation regulation of CSRP1 gene. For example, it has been reported that the methylation of CSRP1 in HCC was elevated, which was consistent with the finding that CSRP1 expression was downregulated in more than half HCC samples [8].

Our study initially provided evidence that CSRP1-knockdown suppressed COAD progression both in vitro and in vivo, suggesting that targeting CSRP1 may be a novel therapeutic direction for COAD treatment. Interestingly, it has been reported that celecoxib treatment induced an upregulated CSRP1 in gastric cancer cells [18], highlighting its crosstalk with antitumor drugs. Therefore, development of CSRP1 inhibitors may provide new insights in cancer treatment.

## 5. Conclusions

High expression of CSRP1 in COAD tissues indicates unfavorable disease prognosis through promoting COAD proliferation. Knockdown of CSRP1 attenuates COAD growth both in vitro and in vivo.

## Figures and Tables

**Table 1:** Correlations between CSRP1 expression and COAD patients' characteristics.

Variables	Cases (*n* = 167)	CSRP1 protein level		*P* value
		Low (*n* = 90)	High (*n* = 77)	
Age (years)				
≤65	80	43	37	0.972
>65	87	47	40	
Sex				
Female	86	52	34	0.079
Male	81	38	43	
Tumor location				
Ascending colon	43	24	19	0.227
Transverse colon	30	20	10	
Descending-sigmoid colon	94	46	48	
Tumor size (cm)				
≤3.5	86	56	30	0.003^*∗∗*^
>3.5	81	34	47	
*T* stage				
*T*1	33	24	9	0.058
*T*2	21	8	13	
*T*3	91	48	43	
*T*4	22	10	12	
Lymph node status				
Negative	96	58	38	0.049^*∗*^
Positive	71	32	39	
Chemotherapy				
No or unknown	109	61	48	0.462
Accepted	58	29	29	

**Table 2:** Kaplan-Meier overall survival analyses of enrolled COAD patients.

Variables	Cases (*n* = 167)	Survival months (mean ± S.D.)	5-year OS (%)	*P* value
Age (years)				
≤65	80	74.4 ± 3.4	77.9	0.001^*∗∗*^
>65	87	58.6 ± 4.1	50.7	
Sex				
Female	86	69.9 ± 3.9	66.2	0.408
Male	81	58.1 ± 3.3	60.7	
Tumor location				
Ascending colon	43	73.5 ± 4.6	72.7	0.190
Transverse colon	30	58.1 ± 6.4	52.7	
Descending-sigmoid colon	94	65.5 ± 3.9	62.9	
Tumor size (cm)				
≤3.5	86	67.8 ± 4.0	65.7	0.579
>3.5	81	63.7 ± 4.0	61.4	
*T* stage				
*T*1	33	75.3 ± 5.9	70.0	0.007^*∗∗*^
*T*2	21	65.0 ± 6.5	63.6	
*T*3	91	67.4 ± 3.5	67.7	
*T*4	22	43.0 ± 7.6	35.2	
Lymph node status				
Negative	96	73.2 ± 3.3	66.9	0.017^*∗*^
Positive	71	56.0 ± 4.2	60.3	
Chemotherapy				
No or unknown	109	64.0 ± 3.5	56.6	0.088
Accepted	58	69.0 ± 3.9	78.1	
CSRP1 protein level				
Low	90	73.4 ± 3.5	71.6	0.014^*∗*^
High	77	58.7 ± 4.4	53.3	

**Table 3:** Multivariate analysis for overall survival of enrolled COAD cohort.

Variables	HR	95% CI	*P* value
Age (years)			
≤65	Reference		
>65	2.489	1.337–4.632	0.004^*∗∗*^
*T* stage			
*T*1	Reference		
*T*2	0.982	0.337–2.861	0.974
*T*3	1.098	0.460–2.621	0.834
*T*4	3.108	1.123–8.600	0.029^*∗*^
Lymph node status			
Negative	Reference		
Positive	2.853	1.501–5.424	0.001^*∗∗*^
Chemotherapy			
No or unknown	Reference		
Accepted	0.339	0.165–0.695	0.003^*∗∗*^
CSRP1 protein level			
Low	Reference		
High	1.895	1.078–3.330	0.026^*∗*^

## Data Availability

The original data used to support the findings of this study are available from the corresponding author upon request.
